# Altered alcohol consumption during COVID-19 pandemic lockdown

**DOI:** 10.1186/s12937-021-00699-0

**Published:** 2021-05-11

**Authors:** Julius Steffen, Jenny Schlichtiger, Bruno C. Huber, Stefan Brunner

**Affiliations:** 1 Department of Medicine I, Ludwig-Maximilians-University Munich, University Hospital, Ziemssenstrasse 1, 80336 Munich, Germany; 2 DZHK (German Centre for Cardiovascular Research), Partner Site Munich, Munich Heart Alliance (MHA), Marchioninistrasse 15, 81377 Munich, Germany

**Keywords:** SARS-CoV-2, Alcohol misuse, Lifestyle, Prevention

## Abstract

**Background:**

Since the onset of the COVID-19 pandemic in December 2019, many countries around the world have imposed lockdown measures in order to reduce virus spread. Social isolation is known to have a significant psychological impact, potentially triggering alcohol misuse in adults. In our study, we aimed to investigate the effect of COVID-19 lockdown measures on alcohol consumption in adults in Bavaria.

**Methods:**

In this cross-sectional study, we enrolled 2067 participants, with 1961 young adults (mean age 23.3 ± 4.1) and 106 mature adults (mean age 66.7 ± 9.7). Participants were asked to complete a standardized questionnaire, semi-quantitatively evaluating the alcohol drinking behaviour before and during the pandemic lockdown.

**Results:**

After implementation of lockdown, the alteration of alcohol consumption was significantly different between young and mature adults (*p* <  0.001). Among young adults, 42% reported unchanged drinking behaviour compared to 76% in the mature adult group; 44% of young adults reported to drink less compared to only 7% of mature adults. An increase in alcohol consumption was only reported by 14% of young adults and 17% of mature adults. Interestingly, in the entire cohort, the change of alcohol intake was most pronounced among moderate drinkers (> 0 to < 5 drinks/week) in both age groups (*p* <  0.001). Ordinal logistic regression revealed female sex, low BMI and younger age to be associated with a decrease in number of self-reported drinks/week.

**Conclusion:**

The COVID-19 pandemic lockdown significantly affected alcohol drinking behaviour. Further studies exploring long-term effects on potential alcohol misuse and the relevance on public health are warranted.

**Trial registration:**

The study was retrospectively registered at ClinicalTrials.gov (NCT04361877) on April 24, 2020.

**Supplementary Information:**

The online version contains supplementary material available at 10.1186/s12937-021-00699-0.

## Introduction

**Fig. 1:**
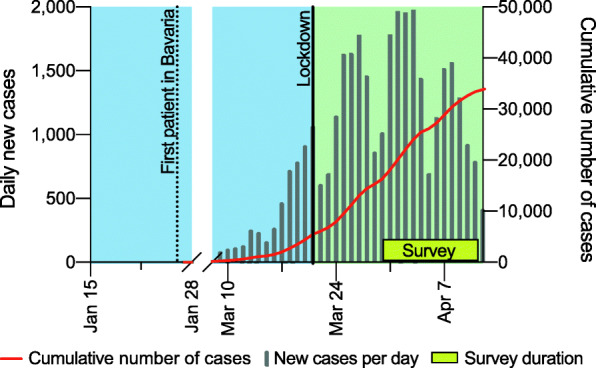
Daily new cases in Bavaria. Timeline showing the number of new confirmed infections with SARS-CoV-2 in Bavaria per day (grey bars) and the cumulative number of cases (red line). The first confirmed case of COVID-19 in Bavaria was on the 27th of January (dotted line), which was also the first case in Germany. Lockdown was implemented by the local authorities in Bavaria on the 21st of March (line). Study participants were asked to compare their alcohol consumption habits before the lockdown (pastel blue area) to during the study period after implementation of the lockdown (pastel green area). The yellow box indicates the data collection period of the questionnaire

Since the onset of the COVID-19 pandemic in December 2019, many countries around the world have imposed lockdown measures in order to reduce virus spread. In the German federal state of Bavaria, lockdown was implemented on the 21st of March by the local government (Fig. 1). Restrictions were similar to many other regions and prohibited visits to restaurants, bars, cafes, and beer gardens. Exceptions to the curfew were going to work, necessary shopping, or visits to doctors and pharmacies [1].

Social isolation is known to have a significant psychological impact, potentially triggering alcohol misuse in adults [2, 3]. According to the World Health Organization, alcohol misuse usually contributes to more than 5% of global disease burden [4]. In the context of COVID-19, patients with alcohol use disorder or alcohol-associated liver disease and attributable comorbidities have an increased risk [2, 5]. The impact of long-term social isolation during the COVID-19 pandemic lockdowns on the level of alcohol consumption and its relevance for public health was recently discussed by Clay and Parker [6].

Two different scenarios how COVID-19 pandemic lockdowns could affect alcohol consumption have been suggested in the literature [7]. First, limited availability, tight budgets, and government restrictions would lead to a reduction in alcohol use. Second, stress and anxiety due to fear of infection, unemployment, or social isolation can trigger psychological distress mechanisms resulting in increased alcohol use [8].

In our study, we hypothesised that due to these scenarios, young people would drink less than mature people during the pandemic. We therefore aimed to investigate the effect of lockdown measures on alcohol consumption in young and mature adults in Bavaria.

## Methods

In order to assess the change in alcohol consumption during lockdown, we conducted a large-scale survey among young adults and mature adults. Data for young adults (up to 50 years of age) were collected within the online cross-sectional COLA (COVID-19 Pandemic Lockdown in Young Adults) study, registered at clinicaltrials.gov (NCT04361877). The survey invitation was emailed to students who consented to receive survey invitations at LMU Munich and 5 other Bavarian universities. Data for mature adults (older than 50 years) were collected in a similar online and print questionnaire, sent to randomly chosen mature adults from diverse educational and financial backgrounds in different regions of Bavaria. These included family members of hospital co-workers (e.g. doctors, nurses, physiotherapists, technicians, secretaries), who then passed on printouts to friends. A power calculation has not been performed prior to study conductance.

Participants were asked to provide demographic data (age, weight, height, educational level) and to compare their alcohol consumption during lockdown to before on a three-level scale (“more”, “less”, “unchanged”). Also, they semi-quantified the number of drinks per week before, and after, lockdown implementation on an ordinal scale (“0 drinks”, “0–2 drinks”, “2–5 drinks”, or “> 5 drinks” per week), with 1 drink corresponding to 500 ml of beer, 100 ml of wine, or 20 ml of liquors. See appendix for more details.

All data sets with information on age and results on semi-quantified number of drinks were included in the analysis. All data collection was performed in accordance with the Declaration of Helsinki. The study was approved by the ethics committee of the Ludwig-Maximilians-University (LMU) Munich, Germany (approval number 20–268 KB). Participants were asked to report about their alcohol drinking behaviour before and during lockdown (Fig. 1).

Shapiro-Wilk test was applied for normality assessment. Differences between groups were evaluated using Kruskal-Wallis-Test for continuous data and Chi^2^-Test (Pearson Chi-Square) for ordinal or nominal data. Three different univariate and multivariate logistic regression analyses were performed to identify the protective factors and risk factors for alterations in alcohol consumption during lockdown compared to before, i.e. for i) drinking more, ii) drinking less, or iii) drinking over 5 drinks per week. Additionally, an ordinal regression model was used with the drinks category being the dependent variable and age group (≤ 50 years regarded as “young”, > 50 years regarded as “mature”), gender, BMI group (i.e. BMI < 20, BMI ≥ 20 and ≤ 25 [reference], and BMI > 25 kg/m^2^), and a high education level (i.e. having acquired Abitur or university degree) serving as independent variables.

A *p*-value < 0.05 was regarded statistically significant for all tests. Continuous data are depicted as mean ± standard deviation or median [interquartile range (IQR)]. Statistical analysis was performed using R (RStudio version 1.2.5033).

## Results

A total of 2070 participants completed the questionnaires, with 1961 young adults (50 years or younger) and 106 mature adults (over 50 years) included in the analysis. Return rate for online and paper-based survey were 24% (1980 out of 8252) and 41% (77 out of 187), respectively. Mean age was 23.3 ± 4.1 and 66.7 ± 9.7, respectively, and 71.4% (young adults, *n* = 1385) vs. 58.1% (mature adults, *n* = 61) were female. Body mass index was significantly higher in the older group (25.7 [23.4–28.3] vs. 21.6 [20.1, 23.4] kg/m^2^) (Table 1). No participant was below the legal drinking age. Results stratified by gender or BMI groups are shown in Supplementary Table S1 and S2.

**Table 1:** Baseline Characteristics

	Total (*N* = 2067)	Mature (*N* = 106)	Young (*N* = 1961)	*p* value
Female	1446 (70.7%)	61 (58.1%)	1385 (71.4%)	< 0.01
Age (years)	25.6 ± 10.6	66.7 ± 9.7	23.4 ± 4.1	< 0.01
BMI (kg/m^2^)	21.7 [20.2–23.8]	25.7 [23.4–28.3]	21.6 [20.1–23.4]	< 0.01
BMI groups				
BMI ≥20 and ≤ 25 kg/m^2^	1273 (62.0%)	37 (34.9%)	1236 (63.5%)	< 0.01
BMI > 25 kg/m^2^	320 (15.6%)	62 (58.5%)	258 (13.3%)	
BMI < 20 kg/m^2^	459 (22.4%)	7 (6.6%)	452 (23.2%)	
Highest educational degree				
Not finished school	1 (0.0%)	1 (0.9%)	0 (0.0%)	< 0.01
Basic secondary school	18 (0.9%)	18 (17.0%)	0 (0.0%)	
Intermediate secondary school	31 (1.5%)	30 (28.3%)	1 (0.1%)	
Abitur (qualification for university) ^a^	1976 (95.6%)	16 (15.1%)	1960 (99.9%)	
Apprenticeship	17 (0.8%)	17 (16.0%)	0 (0.0%)	
University degree^a^	24 (1.2%)	24 (22.6%)	0 (0.0%)	
Alcohol amount since lockdown implementation				
less	824 (40.2%)	7 (6.7%)	817 (42.0%)	< 0.01
unchanged	934 (45.5%)	80 (76.2%)	854 (43.9%)	
more	293 (14.3%)	18 (17.1%)	275 (14.1%)	

*BMI* Body mass index. All numbers are given as median [inter-quartile range], mean ± standard deviation or total number and percentage of group. ^a^Abitur and university degree were regarded as high education level in further analyses

**Fig. 2:**
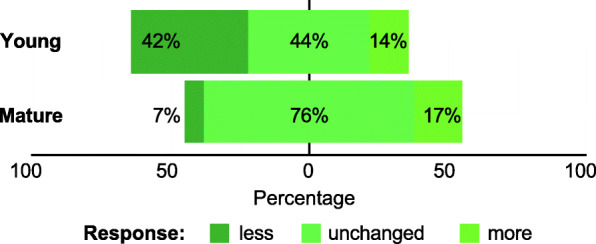
Change in alcohol consumption since lockdown implementation. Participants were asked if their alcohol consumption had changed since implementation of lockdown measures. 44% (*n* = 854) of young adults and 76% (*n* = 80) of mature adults reported to have an unchanged drinking behaviour. More young adults (42%, *n* = 817) than mature adults (7%, *n* = 7) stated to be drinking less. The number of participants drinking more was similar in both age groups (mature adults, 17%, *n* = 18, vs. young adults, 14%, *n* = 275, *p* < 0.001 for all groups)

The fraction of participants from the two age groups reporting to be consuming more, less, or an unchanged amount of alcohol since the implementation of lockdown were significantly different (*p* <  0.001, Fig. 2). 44% (*n* = 854) of young adults reported to not have changed their drinking behaviour compared to 76% (*n* = 80) of mature adults. The fraction of participants stating to be drinking less was larger among the young adults (42%, *n* = 817) compared to mature adults (7%, *n* = 7), while only 14% (young adults, *n* = 275) and 17% (mature adults, *n* = 18) consume more alcohol.

**Fig. 3:**
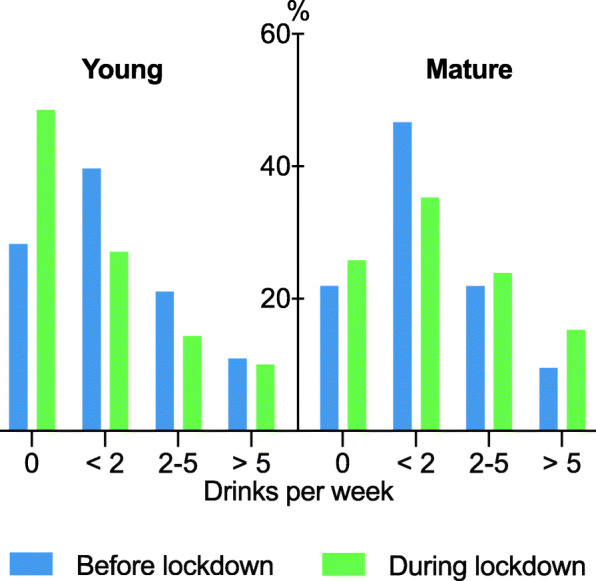
Quantification of drinking behaviour before and during lockdown. Participants were asked to semi-quantify the number of drinks per week they consumed before and during lockdown. Among young adults, a marked increase in participants stating to be drinking 0 drinks per week was observed while all numbers in all other categories decreased slightly. Mature adults did not change their drinking behaviour as much

Before lockdown, most participants reported to consume up to 2 drinks/week (Fig. 3). Generally, only a small fraction of participants drank 2–5 or more than 5 drinks/week, with similar rates in both age groups. However, during lockdown, most young adults consumed 0 drinks/week and the number of people in all other categories decreased. Contrasting this, only slight changes were found among mature adults, with a trend towards more participants in the 2–5 drinks/week and more than 5 drinks/week groups.

More than half of the participants with more frequent consumption of alcoholic beverages (more than 2 drinks/week) from the young adults group decreased their alcohol consumption (55%, *n* = 341) (Supplementary Table S3). Among the frequent drinkers in the mature age group (*n* = 33), only 1 participant (3%) had a decreased alcohol consumption (Supplementary Table S4). Due to limited group size, more detailed analyses evaluating associated factors for this were not possible.

Univariate analyses indicated BMI groups (BMI < 20 kg/m^2^ or BMI > 25 kg/m^2^), age, abstinence (i.e. 0 drinks/week) and frequent drinking (more than 2 drinks/week) were indicators for drinking more or less during lockdown. However, in multivariate logistic regression models for drinking more or less during lockdown respectively, after adjustment for interactions, pre-lockdown abstinence (0 drinks/week) was the only factor protecting against drinking more (odds ratio, OR 0.24, 95% confidence interval, CI [0.16–0.35], Table 2) and young age was the only associated factor for drinking less (OR 9.91, 95% CI [4.92–23.8], Table 3). Female sex was found to be protecting from being a heavy drinker (> 5 drinks/week) during lockdown (OR 0.43, 95% CI [0.32–0.57], Table 4). Educational levels and BMI did not serve as indicators in the multivariate regression models evaluated.

**Table 2:** Univariate analysis and multivariate regression model for “drinking more”

Univariate analyses					Multivariate regression model	
	False (***n*** = 1760)	True (***n*** = 293)	Odds ratio [95% CI]	***P*** value	Odds ratio [95% CI]	***P*** value
**Age group**						
Mature	87 (4.95%)	18 (6.14%)	Reference	Reference		
Young	1671 (95.1%)	275 (93.9%)	0.79 [0.48–1.38]	0.390		
**Gender**						
M	497 (28.5%)	96 (33.2%)	Reference	Reference		
F	1244 (71.5%)	193 (66.8%)	0.80 [0.62–1.05]	0.109		
**High education level**	1706 (96.9%)	281 (95.9%)	0.73 [0.40–1.46]	0.359		
**BMI group**						
normal	1090 (62.3%)	175 (60.6%)	Reference	Reference		
BMI > 25 kg/m^2^	259 (14.8%)	58 (20.1%)	1.40 [1.00–1.93]	**0.049**	1.351 [0.965–1.872]	0.075
BMI < 20 kg/m^2^	400 (22.9%)	56 (19.4%)	0.87 [0.63–1.20]	0.407	0.948 [0.679–1.307]	0.749
**Abstinent before**	536 (30.6%)	28 (9.56%)	0.24 [0.16–0.35]	**< 0.001**	0.248 [0.162–0.365]	**< 0.001**

Table legend: *CI* Confidence interval. High education level was defined as Abitur (highest school degree in Germany) or university degree. *BMI* Body-mass index, *M* Male, *F* Female gender. A BMI between 20 and 25 kg/m^2^ was regarded normal in this analysis

**Table 3:** Univariate analysis and multivariate regression model for “drinking less”

Univariate analyses					Multivariate regression model	
	False (n = 1760)	True (n = 293)	Odds ratio [95% CI]	***P*** value	Odds ratio [95% CI]	***P*** value
**Age group**						
Mature	98 (7.99%)	7 (0.85%)	Reference	Reference		
Young	1129 (92.0%)	817 (99.2%)	9.91 [4.92–23.8]	**< 0.001**	9.619 [4.734–23.086]	**< 0.001**
**Gender**						
M	363 (29.8%)	230 (28.3%)	Reference	Reference		
F	854 (70.2%)	583 (71.7%)	1.08 [0.89–1.31]	0.456		
**High education level**	1166 (95.0%)	821 (99.5%)	10.5 [4.31–35.4]	**< 0.001**		
**BMI group:**						
normal	740 (60.6%)	525 (64.3%)	Reference	Reference		
BMI > 25 kg/m^2^	212 (17.4%)	105 (12.9%)	0.70 [0.54–0.90]	**0.006**	0.884 [0.673–1.157]	0.371
BMI < 20 kg/m^2^	269 (22.0%)	187 (22.9%)	0.98 [0.79–1.22]	0.856	0.965 [0.775–1.201]	0.753

Table legend: *CI* Confidence interval. High education level was defined as Abitur (highest school degree in Germany) or university degree. Education level was not included in the multivariate analysis due to interaction with age group. *M* Male, *F* Female gender, *BMI* Body-mass index. A BMI between 20 and 25 kg/m^2^ was regarded normal in this analysis

**Table 4:** Univariate analysis and multivariate regression model for “drinking >5 drinks/week”

Univariate analyses					Multivariate regression model	
	False (n = 1760)	True (n = 293)	Odds ratio [95% CI]	***P*** value	Odds ratio [95% CI]	***P*** value
**Age group**						
Mature	89 (4.83%)	16 (7.55%)	Reference	Reference		
Young	1755 (95.2%)	196 (92.5%)	0.62 [0.36–1.11]	0.104		
**Gender**						
M	497 (27.2%)	98 (46.7%)	Reference	Reference		
F	1328 (72.8%)	112 (53.3%)	0.43 [0.32–0.57]	**< 0.001**	0.469 [0.347–0.635]	**< 0.001**
**High education level**	1787 (96.8%)	204 (96.2%)	0.83 [0.41–1.91]	0.633		
**BMI group:**						
normal	1131 (61.7%)	138 (65.4%)	Reference	Reference		
BMI > 25 kg/m^2^	274 (14.9%)	42 (19.9%)	1.26 [0.86–1.81]	0.230	1.158 [0.787–1.673]	0.444
BMI < 20 kg/m^2^	428 (23.3%)	31 (14.7%)	0.60 [0.39–0.88]	**0.009**	0.729 [0.473–1.092]	0.137

Table legend: *CI* Confidence interval. High education level was defined as Abitur (highest school degree in Germany) or university degree. *M* Male, *F* Female gender, *BMI* Body-mass index. A BMI between 20 and 25 kg/m^2^ was regarded normal in this analysis

Additionally, an ordinal logistic regression model was performed to find factors associated with a decrease to a lower semi-quantitative category for number of drinks per week. Three factors could be identified for this: young vs. mature age (odds ratio, OR, 0.42 [95% confidence interval, CI, 0.24–0.74], *p* = 0.003), female vs. male gender (OR, 0.69 [95% CI, 0.57–0.83], *p* <  0.001), and BMI < 20 kg/m^2^ vs. BMI ≥ 20 and ≤ 25 kg/m^2^ (OR 0.72 [95% CI 0.58–0.89], *p* = 0.002) were found to be associated with a decrease to a lower category for number of drinks per week. A BMI > 25 kg/m^2^ or a high education level had no statistically significant effects (Supplementary Table S5).

## Discussion

In summary, we conducted a regional cross-sectional study in Bavaria, Germany, and found a significant age-dependent change in overall alcohol consumption. Young adults appear to reduce (42%) or not alter (44%) their drinking habits. In contrast, only 7% of mature adults reduced their alcohol consumption, and in 17%, an increase was observed. Most mature adults (76%) did not change their drinking habits.

The self-reported levels of alcohol consumption are comparable to the literature on alcohol consumption in young [9] or mature adults [10]. Generally, Germany ranks among the countries with the highest alcohol consumption worldwide [4].

Our results on changes in drinking behaviour are in accordance with the proposed scenario of a decline in alcohol consumption during the early phase of the crisis [7]. The effect was mainly limited to young adults, possibly because they tend to live in student dorms or single studios and consume alcohol mainly when meeting friends in bars, restaurants, or at private parties [11]. This effect was also observed in South Africa, where strict alcohol control policies have been implemented in order to prevent alcohol-related accidents [7].

Perhaps, in mature adults, the lack of drinking in bars or restaurants was counter-balanced by home drinking [2]. Although the pandemic leads to high stress levels among survivors [3] we did not observe an increased alcohol consumption as self-medication for depression or anxiety during the pandemic. Interestingly, in our analysis, the effect of female gender on alcohol use was not as strong as literature might suggest. Men are more prone for psychological distress, possibly leading to increased drinking [8].

In terms of prevention of chronic alcohol-related morbidities, the results are somewhat satisfactory for the moment, and a spike of drinking disorders or alcohol dependency does not seem likely. However, an economic crisis as a result of the current pandemic could, in the medium-term, lead to the proposed alternative scenario with high unemployment rates and an increased alcohol consumption due to more free time [2].

### Limitations

This study has limitations and is subject to different types of bias. It is a cross-sectional online-based survey and results cannot be reassessed in the future. Data and conclusions drawn are based on self-reported levels of alcohol consumption, which may be affected by information bias. Data were collected at only one single time point, and people were asked to provide their self-assessed consumption amount before lockdown retrospectively. It was object to selection bias, with an unsystematic distribution of the questionnaire and low return rates, and instrument bias, as there were a paper-based and an online version of the questionnaire. Most factors evaluated in the models interacted with the age group since they were not evenly distributed between groups. This and the low number of participants in the mature adults group limit the generalizability of the results mainly to students in Bavaria.

## Conclusion

In conclusion, we show that alcohol consumption in Bavaria, Germany, during COVID-19 lockdown is altered in an age-dependent manner. Although an increase of drinking was not observed right now, further studies exploring regional differences and long-term effects are warranted.

## Supplementary Information

**Additional file 1: Table S1.** Results by BMI group. **Table S2.** Results by gender. **Table S3.** Alcoholic beverages per week in young adults. **Table S4.** Alcoholic beverages per week in mature adults. **Table S5.** Ordinal logistic regression model for drinking less during lockdown.

**Additional file 2.** Online Questionnaire (extract, translation).

## Data Availability

The datasets used and/or analysed during the current study are available from the corresponding author on reasonable request.

## Abbreviations

BMI: Body mass index
CI: Confidence interval
COVID-19: Coronavirus disease 2019
OR: odds ratio
