# Supplementary material for: Altered alcohol consumption during COVID-19 pandemic lockdown
Source: Nutr J. 2021 May 11;20:44. doi: 10.1186/s12937-021-00699-0 (PMC8112739; doi:10.1186/s12937-021-00699-0)
Supplement: Supplementary file 1 — Additional file 1: Table S1. Results by BMI group. Table S2. Results by gender. Table S3. Alcoholic beverages per week in young adults. Table S4. Alcoholic beverages per week in mature adults. Table S5. Ordinal logistic regression model for drinking less during lockdown. [file 12937_2021_699_MOESM1_ESM.docx]

Altered alcohol consumption during COVID-19 pandemic lockdown

Julius Steffen, MD, Jenny Schlichtiger, MSc, Bruno C. Huber, MD, Stefan Brunner, MD

**Supplementary Tables**

**Supplementary Table S1.** Results by BMI group

|  | **Normal weight (N=1274)** | **BMI >25 kg/m^2^ (N=320)** | **BMI <20 kg/m^2^ (N=460)** |  |
| --- | --- | --- | --- | --- |
| **sex** | 850 (67.6%) | 171 (54.5%) | 416 (90.6%) |  |
| **Age (years)** | 24.6 ±8.5 | 32.8 ±17.8 | 23.3 ±6.4 |  |
| **BMI (kg/m^2^)** | 21.9 [21.0-23.1] | 27.0 [25.7-28.9] | 19.0 [18.4-19.6] |  |
| **Change of alcohol consumption** | | | | |
| less | 525 (41.5%) | 105 (33.1%) | 187 (41.0%) |  |
| unchanged | 565 (44.7%) | 154 (48.6%) | 213 (46.7%) |  |
| more | 175 (13.8%) | 58 (18.3%) | 56 (12.3%) |  |
| **No of drinks/week before lockdown** | | | | |
| 0 | 339 (26.7%) | 71 (22.5%) | 161 (35.0%) |  |
| 0-2 | 493 (38.9%) | 141 (44.6%) | 184 (40.0%) |  |
| 2-5 | 287 (22.6%) | 69 (21.8%) | 76 (16.5%) |  |
| >5 | 149 (11.8%) | 35 (11.1%) | 39 (8.5%) |  |
| **No. of drinks/week during lockdown** | | | | |
| 0 | 578 (45.5%) | 128 (40.5%) | 260 (56.6%) |  |
| 0-2 | 367 (28.9%) | 90 (28.5%) | 107 (23.3%) |  |
| 2-5 | 186 (14.7%) | 56 (17.7%) | 61 (13.3%) |  |
| >5 | 138 (10.9%) | 42 (13.3%) | 31 (6.8%) |  |

BMI, body mass index. All numbers are given as median [inter-quartile range], mean ± standard deviation or total number and percentage of group.

**Supplementary Table S2.** Results by gender

|  | **Male (N=598)** | **Female (N=1448)** |
| --- | --- | --- |
| **Age (years)** | 26.9 ±12.3 | 25.0 ±9.8 |
| **BMI** | 22.9 [21.6-24.9] | 21.1 [19.7-23.0] |
| **BMI group** |  |  |
| Normal weight | 408 (68.7%) | 850 (59.2%) |
| BMI >25 kg/m^2^ | 143 (24.1%) | 171 (11.9%) |
| BMI <20 kg/m^2^ | 43 (7.2%) | 416 (28.9%) |
| **Change of alcohol consumption** | | |
| less | 230 (38.8%) | 583 (40.6%) |
| unchanged | 267 (45.0%) | 661 (46.0%) |
| more | 96 (16.2%) | 193 (13.4%) |
| **No of drinks/week before lockdown** | | |
| 0 | 134 (22.6%) | 436 (30.3%) |
| 0-2 | 210 (35.4%) | 606 (42.1%) |
| 2-5 | 140 (23.6%) | 289 (20.1%) |
| >5 | 110 (18.5%) | 110 (7.6%) |
| **No. of drinks/week during lockdown** | | |
| 0 | 241 (40.5%) | 723 (50.2%) |
| 0-2 | 161 (27.1%) | 398 (27.6%) |
| 2-5 | 95 (16.0%) | 207 (14.4%) |
| >5 | 98 (16.5%) | 112 (7.8%) |

BMI, body mass index. All numbers are given as median [inter-quartile range], mean ± standard deviation or total number and percentage of group.

**Supplementary Table S3.** Alcoholic beverages per week in young adults.

|  |  | **During lockdown** | | | |
| --- | --- | --- | --- | --- | --- |
|  | **Drinks / week** | **0** | **< 2** | **2-5** | **> 5** |
| **Before**  **lockdown** | **0** | 511 (93.1%) | 32 (5.8%) | 5 (0.9%) | 1 (0.2%) |
|  | **< 2** | 329 (42.6%) | 301 (38.9%) | 122 (15.8%) | 21 (2.7%) |
|  | **2-5** | 80 (19.5%) | 144 (35.0%) | 110 (26.8%) | 77 (18.7%) |
|  | **> 5** | 25 (11.7%) | 50 (23.4%) | 42 (19.6%) | 97 (45.3%) |

Participants were asked how many drinks per week they consumed before and during lockdown. Combination of the two answers in the young adults group are shown in the table as total number and fraction during lockdown of the before lockdown category.

**Supplementary Table S4.** Alcoholic beverages per week in mature adults.

|  |  | **During lockdown** | | | |
| --- | --- | --- | --- | --- | --- |
|  | **Drinks / week** | **0** | **< 2** | **2-5** | **> 5** |
| **Before**  **lockdown** | **0** | 23 (100%) | 0 (0%) | 0 (0%) | 0 (0%) |
|  | **< 2** | 3 (6.1%) | 37 (75.5%) | 8 (16.3%) | 1 (2.0%) |
|  | **2-5** | 1 (4.3%) | 0 (0%) | 17 (73.9%) | 5 (21.7%) |
|  | **> 5** | 0 (0%) | 0 (0%) | 0 (0%) | 10 (100%) |

Participants were asked how many drinks per week they consumed before and during lockdown. Combination of the two answers in the mature adults group are shown in the table as total number and fraction during lockdown of the before lockdown category.

**Supplementary Table S5.** Ordinal logistic regression model for drinking less during lockdown

| **Variable** | **Odds ratio [95% CI]** | **P value** |
| --- | --- | --- |
| **Young vs. mature** | 0.423 [0.241-0.742] | 0.003 |
| **Female vs. male** | 0.688 [0.573-0.827] | <0.001 |
| **BMI > 25 vs. 20-25 kg/m^2^** | 1.092 [0.86-1.385] | 0.468 |
| **BMI < 20 vs. 20-25 kg/m^2^** | 0.72 [0.583-0.888] | 0.002 |
| **High education level** | 1.431 [0.723-2.841] | 0.304 |

CI, confidence interval. BMI, body mass index. High education level was defined as Abitur (highest school degree in Germany) or university degree.
