# Supplementary material for: Altered alcohol consumption during COVID-19 pandemic lockdown
Source: Nutr J. 2021 May 11;20:44. doi: 10.1186/s12937-021-00699-0 (PMC8112739; doi:10.1186/s12937-021-00699-0)
Supplement: Supplementary file 2 — Additional file 2. Online Questionnaire (extract, translation). [file 12937_2021_699_MOESM2_ESM.docx]

**Altered alcohol consumption during COVID-19 pandemic lockdown**

Julius Steffen, Jenny Schlichtiger, Bruno C. Huber, and Stefan Brunner

**Appendix**

**Online Questionnaire (extract, translation)**

| 1. **Gender** | male  female |
| --- | --- |
| 1. **Age** | number |
| 1. **Height** | number in cm |
| 1. **Weight** | number in kg |
| 1. **What is your highest educational level** | Not finished school  Basic secondary school  Intermediate secondary school  Abitur (qualification for university)  Apprenticeship  University degree |
| 1. **How much alcohol do you consume since the implementation of lockdown?** | less  unchanged  more |
| 1. **Please choose: number of alcoholic beverages BEFORE implementation of lockdown** (0,5 l beer = 2 drinks; 0,1 l wine = 1 drink; 0,02 l liquors = 1 drink) | 0 drinks  0-2 drinks  2-5 drinks  >5 drinks |
| 1. **Please choose: number of alcoholic beverages AFTER implementation of lockdown** (0,5 l beer = 2 drinks; 0,1 l wine = 1 drink; 0,02 l liquors = 1 drink) | 0 drinks  0-2 drinks  2-5 drinks  >5 drinks |
